# Mevalonate Biosynthesis Intermediates Are Key Regulators of Innate Immunity in Bovine Endometritis

**DOI:** 10.4049/jimmunol.1501080

**Published:** 2015-12-16

**Authors:** Gareth D. Healey, Christine Collier, Sholeem Griffin, Hans-Joachim Schuberth, Olivier Sandra, David G. Smith, Suman Mahan, Isabelle Dieuzy-Labaye, I. Martin Sheldon

**Affiliations:** * Institute of Life Science, College of Medicine, Swansea University, Swansea SA2 8PP, United Kingdom;; † University of Veterinary Medicine, 30559 Hannover, Germany;; ‡ INRA, UMR 1198 Biologie du Développement et Reproduction, F-78350 Jouy-en-Josas, France;; § Institute of Infection, Immunity and Inflammation, College of Medical, Veterinary and Life Sciences, University of Glasgow, Glasgow G12 8QQ, United Kingdom;; ¶ Moredun Research Institute, Midlothian EH26 0PZ, United Kingdom;; ‖ Zoetis, Kalamazoo, MI 49007; and; # Zoetis, 75014 Paris, France

## Abstract

Metabolic changes can influence inflammatory responses to bacteria. To examine whether localized manipulation of the mevalonate pathway impacts innate immunity, we exploited a unique mucosal disease model, endometritis, where inflammation is a consequence of innate immunity. IL responses to pathogenic bacteria and LPS were modulated in bovine endometrial cell and organ cultures by small molecules that target the mevalonate pathway. Treatment with multiple statins, bisphosphonates, squalene synthase inhibitors, and small interfering RNA showed that inhibition of farnesyl-diphosphate farnesyl transferase (squalene synthase), but not 3-hydroxy-3-methylglutaryl-CoA reductase or farnesyl diphosphate synthase, reduced endometrial organ and cellular inflammatory responses to pathogenic bacteria and LPS. Although manipulation of the mevalonate pathway reduced cellular cholesterol, impacts on inflammation were independent of cholesterol concentration as cholesterol depletion using cyclodextrins did not alter inflammatory responses. Treatment with the isoprenoid mevalonate pathway-intermediates, farnesyl diphosphate and geranylgeranyl diphosphate, also reduced endometrial cellular inflammatory responses to LPS. These data imply that manipulating the mevalonate pathway regulates innate immunity within the endometrium, and that isoprenoids are regulatory molecules in this process, knowledge that could be exploited for novel therapeutic strategies.

## Introduction

**FIGURE 1.**
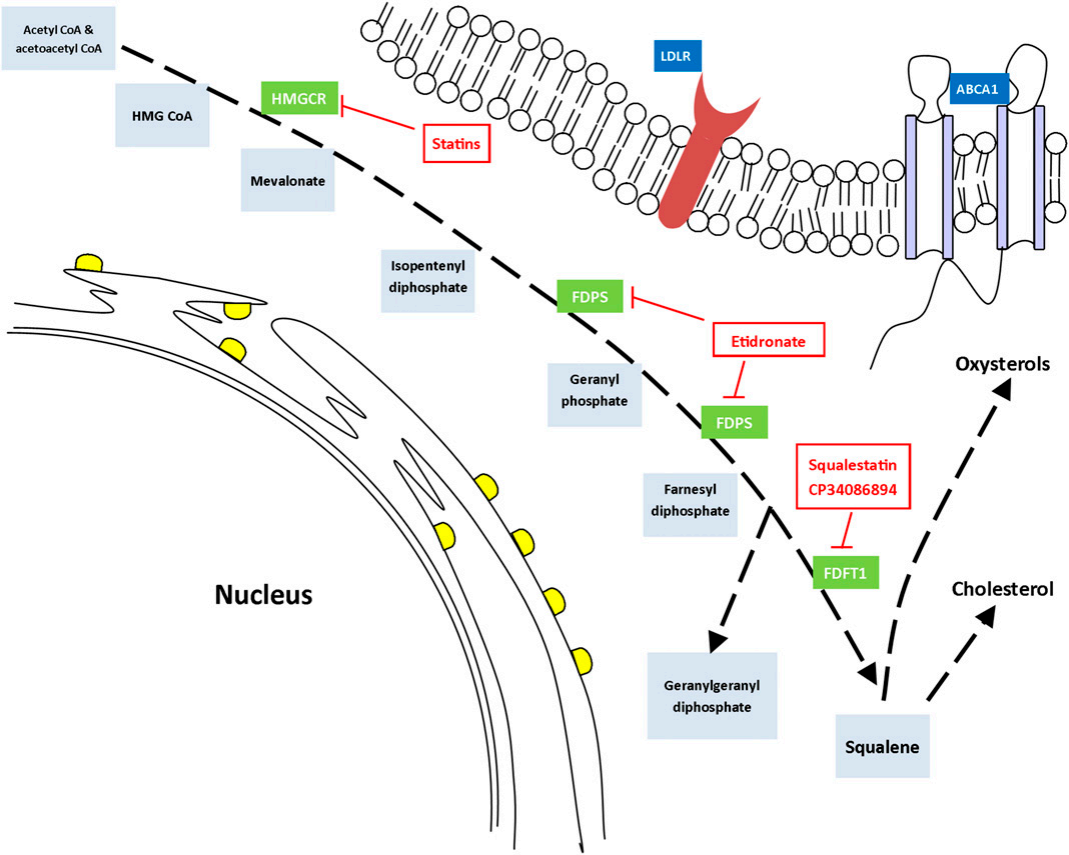
Cholesterol biosynthesis pathway. Cholesterol is the predominant sterol in vertebrates, and in eukaryotes, the mevalonate pathway is the main synthesis pathway for cholesterol. Acetyl CoA and acetoacetyl CoA are converted via the isoprenoids (e.g., FPP and GGPP) to squalene. Three of the key enzymes in this pathway are HMGCR, FDPS, and FDFT1. The importance of cholesterol to a variety of cellular processes means that cholesterol concentration within the cell is tightly regulated. Consequently, cholesterol synthesis is closely linked to cholesterol uptake via receptors such as the low-density lipoprotein receptor (LDLR), and export from the cell via transporters such as the ATP-binding cassette transporter A1 (ABCA1).

Cholesterol is the predominant sterol in vertebrates and it is an essential component of numerous cellular processes. Consequently, cholesterol synthesis, uptake, and efflux are tightly regulated in cells (1). Key to the synthesis of cholesterol is an ancient and diverse family of biological compounds called isoprenoids, which comprises around 30,000 products of the condensation of isopentenyl pyrophosphate and dimethylallyl diphosphate (2). All organisms use these isoprenoid precursors, but they can be synthesized by two independent and nonhomologous pathways, the methylerythritol phosphate and the mevalonate pathways, with the mevalonate pathway dominant in eukaryotes (Fig. 1) (1, 2). Cholesterol and lipid metabolism are essential for normal cellular function, and disruption of mevalonate biosynthesis is associated with diseases such as cancer, autoimmune disease, heart disease, atherosclerosis, and Alzheimer’s disease (3). Key to understanding the importance of mevalonate biosynthesis in disease was seminal work by Goldstein and Brown (1) on the rate-limiting enzyme for cholesterol biosynthesis, 3-hydroxy-3-methylglutaryl CoA reductase (HMGCR), which paved the way for the introduction of statin therapy.

This study used bovine endometritis as a model disease where there is highly localized inflammation, postinfection of the endometrium, initially by pathogenic *Escherichia coli* and then by *Trueperella pyogenes* and other anaerobes in vivo (4–6). The endometritis caused by pathogenic *Escherichia coli* is driven by endometrial epithelial and stromal cell innate immunity, and in particular the sensing of *E. coli* LPS by TLR4, which leads to secretion of the cytokine IL-6 and the chemokine CXCL8 (6, 7). The first objective of this study was to screen modes of action that might modulate endometrial cell inflammatory responses to LPS by using topical administration of small molecules to cells. Several target pathways were identified, but the most striking finding was that modulating the cholesterol synthesis pathway could increase or decrease inflammatory responses to LPS, depending on where in the pathway the inhibitors acted. The demand for metabolisable energy for milk production in postpartum dairy cows leads to significantly increased lipid mobilization, and this lipid mobilization is often associated with metabolic and reproductive disorders, including uterine disease (8). Lower serum cholesterol concentration is also associated with uterine disease in dairy cows (9). However, the mechanisms linking lipid metabolism and uterine disease are not known.

Aiming to understand mevalonate pathway regulation of innate immunity at the endometrial surface, to our knowledge, this is the first comprehensive report to explore the biological link between mevalonate biosynthesis and the occurrence of endometritis. In doing so, this study examined how targeting mevalonate biosynthesis impacted endometrial cell inflammatory responses. We evaluated the effect of manipulating key enzymes of the mevalonate biosynthesis pathway on endometrial cell and ex vivo organ culture (EVOC) responses to LPS, and live *E. coli* and *T. pyogenes*. Inhibition of farnesyl-diphosphate farnesyl transferase (FDFT1; also known as squalene synthase; Fig. 1), which leads to the accumulation of isoprenoids, or treatment with isoprenoids, modulated inflammatory responses. Inhibiting the mevalonate pathway before the synthesis of isoprenoids had little effect on inflammation.

## Materials and Methods

### Cell and organ culture

Uteri with no gross evidence of genital disease or microbial infection were collected from postpubertal mixed-breed beef cattle (*n* = 144 over a 24-mo period) within 15 min of slaughter, as part of the routine operation of a commercial slaughterhouse. Postpartum cattle were not used because of the ubiquitous bacterial contamination and disruption of the epithelium that is typical of the endometrium after parturition (8, 10). The animals were 20- to 26-mo-old, reared on extensive grassland, and had never been pregnant or inseminated. The stage of reproductive cycle was determined by examination of ovarian morphology and vasculature, as described previously, and animals on days 1–4 of the oestrus cycle were used because, similar to postpartum cows, peripheral plasma ovarian hormone concentrations are basal (11). The uteri were kept on ice for ∼1 h until further processing at the laboratory. External surfaces were washed with 70% ethanol, and the uterine horn opened longitudinally with sterile scissors. Because innate immune responses to LPS are the same irrespective of the horn used, one horn was used for the isolation of purified endometrial cell populations and the contralateral horn was used for organ culture (12).

Endometrial cells were isolated as described previously (7, 13). Epithelial and stromal cell populations were distinguished by cell morphology, the presence of cytokeratin and vimentin, respectively, and the absence of immune cell contamination was confirmed by the absence of CD45, as described previously (13, 14). The epithelial and stromal cells were cultured in 1 ml complete medium per well, comprising phenol red–free RPMI 1640 (Sigma-Aldrich, Dorset, U.K.) containing 10% heat-inactivated FBS (Biosera, East Sussex, U.K.), and plated at 1 × 10^5^ cells/ml in 24-well plates (TPP, Trasadingen, Switzerland) ready for treatment. Endometrium was collected using 8-mm diameter punch biopsy, and EVOC was performed as described previously (15). Tissues were cultured in 24-well plates (TPP) containing 2 ml complete medium per well, and treatments were initiated within 4 h of slaughter. During treatment, cells or tissues were maintained in a humidified, 5% CO_2_ in air atmosphere incubator at 37°C.

### Experimental design

#### Treatments.

Cultures of *E. coli* (isolate MS499) (16) or *T. pyogenes* (isolate MS249) (17), previously collected from the uteri of postpartum cows with persistent uterine disease, were grown overnight in Luria-Bertani medium (Sigma-Aldrich) and brain-heart infusion medium (Sigma-Aldrich) supplemented with 5% FBS, respectively, as described previously (6, 18). The concentration of bacteria was measured by colony count and suspended to 1 × 10^8^ CFU/ml in sterile PBS (Life Technologies, Paisley, U.K.), followed by centrifugation at 6000 × *g* for 10 min at 4°C. After washing, bacteria were diluted to 1 × 10^3^ CFU/ml (*E. coli*) or 1 × 10^8^ CFU/ml (*T. pyogenes*) in complete medium. Ultrapure LPS from *E. coli* 0111:B4 was obtained from InvivoGen (Toulouse, France) and used at 100 ng/ml, because this concentration has previously been shown to be optimal for stimulating IL-6 and CXCL8 responses in endometrial cells (14). Full details of the small molecules used as a part of the inflammatory modulator screening are provided in Supplemental Table I. The isoprenoid alcohols farnesol and geranylgeraniol were obtained from Sigma-Aldrich.

### Inflammatory response modulator screening

We selected small molecules (Supplemental Table I) and screened them for their effect on IL-6 secretion from endometrial cells treated with LPS. In brief, endometrial stromal cells were pretreated with control medium or medium containing the small molecule of interest for 24 h, and subsequently challenged with control medium or 100 ng/ml LPS for a further 24 h in control medium or medium containing the small molecule. The supernatants were collected and stored at −20°C before analysis of IL-6 by ELISA. Cell viability was assessed by the mitochondria-dependent reduction of MTT to formazan, as described previously (19). The correlation between MTT OD_570_ measurements and the number of live cells was confirmed using trypan blue exclusion and counting the number of live cells using a hemocytometer.

### Inflammatory responses within bovine endometrial cells/EVOCs

Purified endometrial epithelial (*n* = 9) or stromal (*n* = 9) cells or EVOCs (*n* = 10) were pretreated with control medium or medium containing 10 μM atorvastatin to inhibit HMGCR, 10 μM squalestatin (zaragozic acid) to inhibit FDFT1, or 25 nM dexamethasone as a positive control for 24 h. Endometrial cells were subsequently challenged with control medium or medium containing 100 ng/ml LPS for 24 h, whereas EVOCs were challenged with control medium, or medium containing 1 × 10^3^ CFU/ml *E. coli* or 1 × 10^8^ CFU/ml *T. pyogenes* for 24 h. The supernatants were then collected and stored at −20°C before analysis of IL-6, CXCL8, and IL-1β by ELISA. Cell viability was assessed by MTT as described earlier, and EVOC tissues were weighed to enable IL concentrations to be adjusted for tissue weight.

### Inhibition of the mevalonate pathway and cholesterol sequestration

Purified endometrial epithelial (*n* = 4) and stromal (*n* = 4) cells were pretreated for 24 h with control medium or medium containing a range of concentrations of atorvastatin (0.1–0 μM), etidronate (1–100 μM) to inhibit farnesyl diphosphate synthase (FDPS), squalestatin (0.1–10 μM), or 25 nM dexamethasone. Endometrial cells were subsequently challenged with control medium or medium containing 100 ng/ml LPS for 24 h, in the continued presence of the inhibitors. Supernatants were then collected and stored at −20°C before analysis of IL-6, and CXCL8 by ELISA. Cells were lysed and stored in radioimmunoprecipitation assay buffer (RIPA) buffer at −80°C for analysis of total cell cholesterol by enzymatic assay.

For time-course experiments, purified endometrial stromal cells (*n* = 4) were treated with medium containing 10 μM atorvastatin, 100 μM etidronate, 10 μM squalestatin, or 10 μM CP-34086894 (an alternative inhibitor of FDFT1), for 0, 6, 12, 18, 24, or 48 h. Supernatants were discarded and cells stored in RIPA buffer at −80°C before analysis of total cell cholesterol by enzymatic assay.

For the membrane cholesterol sequestration experiments, purified endometrial stromal cells (*n* = 4) were treated for 0, 1, 6, 12, or 24 h with medium containing 1 mM methyl-β cyclodextrin, which binds to cholesterol. Supernatants were discarded and cells lysed and stored in RIPA buffer at −80°C before analysis of total cell cholesterol by enzymatic assay. To assess the impact of cholesterol reduction on inflammatory responses to LPS, we treated endometrial stromal cells (*n* = 4) with control medium or medium containing 1 mM methyl-β cyclodextrin or 25 nM dexamethasone for 24 h. Cells were then challenged with control medium or 100 ng/ml LPS for 24 h, in the continued presence of the small molecule. Supernatants were collected and stored at −20°C before analysis of IL-6 and CXCL8 by ELISA.

To examine the impact of small molecules on cells, we cultured endometrial cells for 24 h in control medium or medium containing atorvastatin (0.05–10 μM), etidronate (5–200 μM), squalestatin (0.5–20 μM), or methyl-β cyclodextrin (50–2000 μM). Cell viability was assessed by the mitochondria-dependent reduction of MTT to formazan, as described previously (19), and in additional independent experiments by quantification of cellular nucleic acids using the CyQUANT Cell proliferation Assay Kit (ThermoFisher Scientific UK), according to the manufacturer’s instructions.

### Isoprenoids and the regulation of endometrial cell inflammatory responses to LPS

Endometrial epithelial (*n* = 4) and stromal (*n* = 4) cells were pretreated with control medium or medium containing CP-34086894 (0.01–100 μM), squalestatin (0.01–100 μM), the isoprenoids farnesol (0.01–1000 μM) or geranylgeraniol (0.01–1000 μM), or 25 nM dexamethasone for 24 h. Cells were subsequently challenged with 100 ng/ml LPS for 24 h in the continued presence of the small molecule, and supernatants were then collected and stored at −20°C before analysis of IL-6 and CXCL8 by ELISA.

### Enzyme immune assays

Concentrations of IL-1β and IL-6 in cell and EVOC culture supernatants were measured by ELISA, according to the manufacturer’s instructions (Bovine IL-1β Screening Set ESS0027; ThermoFisher Scientific, Perbio Science UK, Cramlington, U.K.; Bovine IL-6 Screening Set ESS0029; ThermoFisher Scientific). Concentrations of CXCL8 in cell and EVOC culture supernatants were measured by the human CXCL8/IL-8 DuoSet ELISA according to the manufacturer’s instructions (DY208; R&D Systems Europe, Abingdon, U.K.), which has previously been validated for the measurement of bovine CXCL8 (20) or by a recently developed bovine CXCL8 ELISA (21). To take into account differences between the weights of EVOC tissues, we report concentrations as picogram per milligram tissue. The limits of detection for IL-1β, IL-6, and CXCL8 were 20.1, 35.6, and 14.3 pg/ml, respectively; the intra-assay coefficients of variance were 4.6, 1.2, and 1.7%, and the interassay coefficients of variance were 7.7, 3.0, and 5.5%, respectively.

Cholesterol concentrations were determined using the Amplex red cholesterol assay kit (Life Technologies). The intra-assay and interassay coefficients of variation were <5%, and the limit of detection was 200 nM.

### Gene expression analysis

Gene expression analysis was performed according to the MIQE guidelines (22). Total RNA was isolated from cells after lysis in RLT buffer using the RNeasy Mini kit (Qiagen, Crawley, U.K.), and reverse transcription of 1 μg mRNA was performed in a 20-μl reaction volume using the QuantiTect RT Kit (Qiagen), according to the manufacturer’s instructions. Quantitative PCR (qPCR) for *HMGCR* and *FDFT1* was performed using SYBR green–based PCR with primers designed using the Eurofins MWG Operon qPCR primer design software (https://ecom.mwgdna.com/services/webgist/dual_probe_design?usca_pZt) and validated by BLAST analysis against the *Bos taurus* (taxid: 9913) Refseq mRNA database. The *HMGCR* and *FDFT1* primers, and *GAPDH* and *ACTB* reference gene primers (12) were obtained from Eurofins MWG Operon and were as follows: *HMGCR* forward, 5′-AGGGAGAACATTGCTCGTGG-3′, reverse, 5′-GTAGTTGGCGAGAACCGACA-3′; *FDFT1* forward, 5′-GGGCACCCTGAGGAGTTCTAC-3′, reverse, 5′-CTCCAGGGAGATCGTTGGGA-3′; *GAPDH* forward, 5′-ATTCCACCCACGGCAAGTTC-3′, reverse, 5′-TCCATCGTCCACCGCAAATGCTTCT-3′; *ACTB* forward, 5′-AAGAAAAAGGGTGTAACGCAG-3′, reverse 5′-TCCATCGTCCACCGCAAATGCTTCT-3′. qPCR was performed in a 25-μl reaction volume comprising 1× QuantiFast SYBR Green PCR Master Mix (Qiagen) with primers added in nuclease-free water to a final concentration of 0.4 mM and 2 μl cDNA. Thermal cycling parameters were: one cycle of 95°C for 5 min, followed by 40 cycles of 95°C for 30 s and 60°C for 60 s. The expression of each gene was normalized against the geometric mean of the two reference genes *GAPDH* and *ACTB*, which were validated as invariant across treatment groups using standard methods (23), and the relative quantification method was used to quantify target gene mRNA within samples as described previously (24). To generate standard curves, we reverse transcribed total RNA extracted from cells to cDNA. Ten-fold serial dilutions of this reference cDNA were prepared (neat to 1 × 10^−5^) in nuclease-free water (Qiagen). For each sample, target and reference gene mRNA abundance was determined from the appropriate standard curve [quantification cycle (Cq)]. Changes in mRNA abundance between samples were then determined from the ratio of the target gene Cq to the reference gene Cq.

### Immunoblotting

Proteins from lysed cells were normalized to 1 μg/μl using the DC Assay (Bio-Rad) and separated (10 μg per lane) using 10% (v/v) SDS-PAGE, with m.w. markers run in parallel lanes (Bio-Rad). After electrophoresis, proteins were transferred to a polyvinylidene difluoride membrane (Bio-Rad); nonspecific sites were blocked using a solution of 5% (w/v) BSA (Sigma-Aldrich) in TBS overnight at 4°C with gentle agitation. Membranes were probed with Abs targeting FDFT1 (NBP1-54855; Novus Biologicals, Cambridge, U.K.), and HMGCR (ab98018; Abcam, Cambridge, U.K.), using Abs selected based on recognition of immunoreactive proteins of appropriate m.w. Primary Abs were used at 1:500 dilutions in 5% (w/v) BSA in TBS for 2 h with gentle agitation. After incubation, membranes were washed three times for 5 min in TBS and 0.1% Tween 20 (pH 7.6). Membranes were then incubated in secondary HRP-conjugated Ab (Cell Signaling Technology, Danvers, MA) in 5% (w/v) BSA in TBS for 1.5 h and washed three times for 5 min in TBS and 0.1% Tween 20 (pH 7.6). Steady-state levels of immunoreactive proteins were visualized using ECL (Western C; Bio-Rad). Protein loading was evaluated and normalized by examining β-actin protein levels using a β-actin Ab (Abcam). The average peak densities of unsaturated bands were analyzed using Quantity-one software (Bio-Rad).

### siRNA

Primary endometrial stromal cells (*n* = 4) were transfected with Lipofectamine RNAiMAX Reagent (Invitrogen) and small interfering RNA (siRNA; designed using Dharmacon siDESIGN Center; Thermo Scientific) targeting *HMGCR* and *FDFT1*. siRNA duplex sequences were siHMG: sense 5′-CAGCAUGGAUAUUGAACAAUU-3′, antisense 5′-UUGUUCAAUAUCCAUGCUGUU-3′; siFDFT1: sense 5′-GCGAGAAGGGAGAGAGUUUUU-3′, antisense 5′-AAACUCUCUCCCUUCUCGCUU-3′. In brief, RNAiMAX-RNAi duplex complexes were formed by adding 50 pmol siRNA to 500 μl Opti-MEM I Reduced Serum Media (without antibiotics; Invitrogen) in each well of a six-well plate (Helena Bioscience). For controls, 50 pmol ONTARGETplus Nontargeting siRNA #1 (Dharmacon) was used instead of the targeted siRNA. Then 7.5 μl RNAiMAX was added to each well containing the diluted RNAi molecules and left for 20 min at room temperature. Exponentially growing cells were then seeded in 2.5 ml complete medium without antibiotics per well to give ∼50% confluence (5 × 10^5^ cells/well). All transfections were carried out in duplicate. Cells were challenged with 100 ng/ml LPS, 24 h after the addition of the siRNA, and changes in mRNA and protein expression were assessed 48 h after transfection.

### Statistical analyses

Statistical analyses were performed using IBM SPSS Statistics 20 with the animal as the experimental unit. Initially the data were tested for homogeneity, and log or square root transformed if appropriate. Data were analyzed by ANOVA and Dunnett’s pairwise multiple comparison *t* test, or by Student *t* test. Data are presented as SEM, a *p* value <0.05 was considered statistically significant, and *n* represents the number of animals.

## Results

### Cholesterol biosynthesis and LPS-mediated inflammation

**FIGURE 2.**
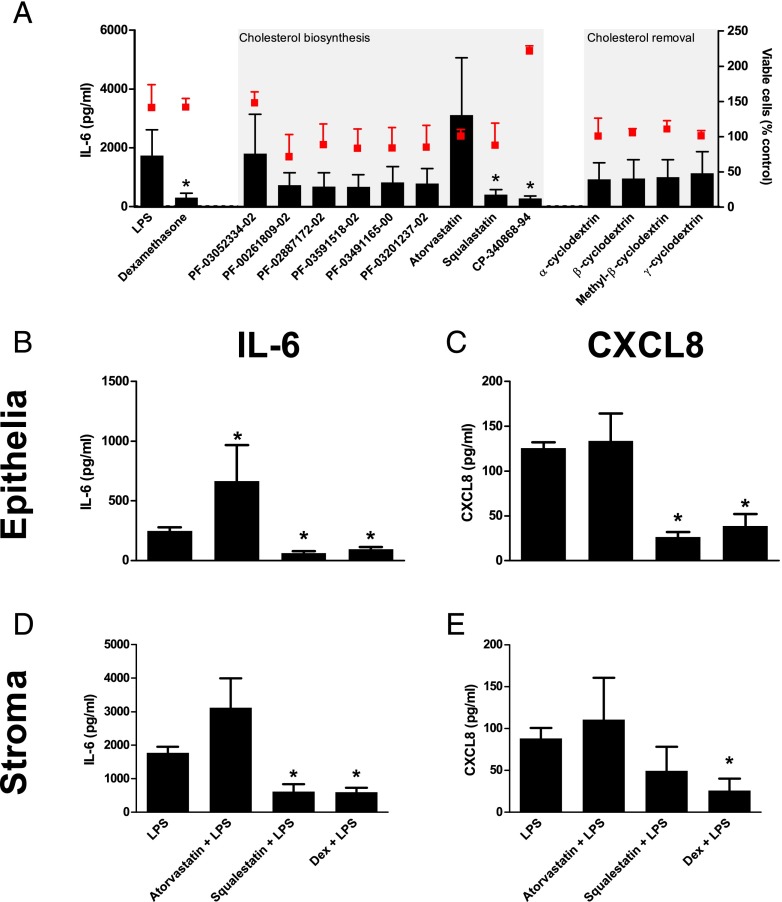
Altered cholesterol biosynthesis modulates inflammatory responses to LPS. Endometrial stromal cells were treated with small molecules from several classes of putative modulators of inflammation for 24 h, before challenge with control medium or 100 ng/ml LPS for 24 h (**A**). Endometrial epithelial (**B** and **C**) or stromal (**D** and **E**) cells were treated with atorvastatin (10 μM), squalestatin (10 μM), or dexamethasone (25 nM, Dex) for 24 h, before challenge with control medium or 100 ng/ml LPS for 24 h. Supernatants were collected and analyzed for IL-6 (A, B, and D) and CXCL8 (C and E) by ELISA (bars), and cell viability was determined by MTT assay (red squares). Data are expressed as mean (SEM) from ≥4 independent experiments. Data were analyzed by ANOVA and Dunnett’s pairwise multiple comparison test; values differ from LPS treatment, *p < 0.05.

We initially explored putative modes of action that might modulate inflammatory responses to LPS. Bovine endometrial stromal cell secretion of IL-6 in response to 24-h treatment with 100 ng/ml LPS is a well-established model of endometritis (7, 14). Primary stromal cells isolated from the uteri of 46 animals were used to screen 49 small molecules (Supplemental Table I). At least four animals were used to test each molecule, and cell viability was determined by MTT assay. Cellular responsiveness to LPS was confirmed in each experiment (control versus LPS 70.6 ± 10.9 versus 1567.9 ± 405.9 pg/ml IL-6, *p* < 0.001). In addition, dexamethasone was used as a positive control because it has a well-established anti-inflammatory effect, which was confirmed in the present screening system (LPS versus Dex + LPS 1567.9 ± 405.9 versus 304.9 ± 62.4 pg/ml IL-6, *p* < 0.001). Molecules of interest were defined by an inflammatory response >1 SD from the reference response to LPS. Based on the IL-6 response and cell viability data presented in Supplemental Table I, JAK-STAT signaling, nuclear receptor signaling, chemokine receptor signaling, and cholesterol biosynthesis were identified as potential targets for therapeutic intervention aimed at reducing cellular inflammatory responses to LPS. Of particular interest were molecules targeting cholesterol or cholesterol biosynthesis (Fig. 1), where there was a differential cellular response to several modulators of the pathway (Fig. 2A). After exposure to LPS, endometrial stromal cells pretreated with squalestatin produced less IL-6 compared with LPS-treated cells, whereas cells treated with atorvastatin increased IL-6 production (Fig. 2A). These data imply that disruption of lipid metabolism and the mevalonate pathway may influence endometrial innate immune inflammatory responses to LPS.

### Cholesterol biosynthesis and inflammatory responses within the bovine endometrium

The differential effect of inhibiting cholesterol biosynthesis on LPS-mediated inflammation was further explored using purified endometrial cell populations and intact endometrial organ cultures. Epithelial cells secreted more IL-6 (control versus LPS 28.2 ± 19.1 versus 246.9 ± 30.8 pg/ml IL-6, *p* < 0.05) and more CXCL8 (control versus LPS 3.1 ± 3.1 versus 125.7 ± 6.4 pg/ml CXCL8, *p* < 0.05) in response to challenge with 100 ng/ml LPS for 24 h, as expected. However, pretreatment of epithelial cells with atorvastatin for 24 h increased IL-6 but not CXCL8 secretion in response to LPS challenge (Fig. 2B, 2C), whereas pretreatment with squalestatin or the positive control dexamethasone reduced the secretion of IL-6 (*p* < 0.05) and CXCL8 (*p* < 0.05). Stromal cells secreted more IL-6 (control versus LPS 32.9 ± 15.4 versus 1765.8 ± 190.4 pg/ml IL-6, *p* < 0.05) and more CXCL8 (control versus LPS 11.6 ± 6.6 versus 88.1 ± 12.5 pg/ml CXCL8, *p* < 0.05) in response to challenge with 100 ng/ml LPS for 24 h. Pretreatment of stromal cells with atorvastatin for 24 h had no significant effect on IL-6 or CXCL8 secretion, but pretreatment with squalestatin or dexamethasone for 24 h reduced the secretion of IL-6 in response to LPS challenge (*p* < 0.05), and dexamethasone also reduced the secretion of CXCL8 (*p* < 0.05; Fig. 2D, 2E).

**FIGURE 3.**
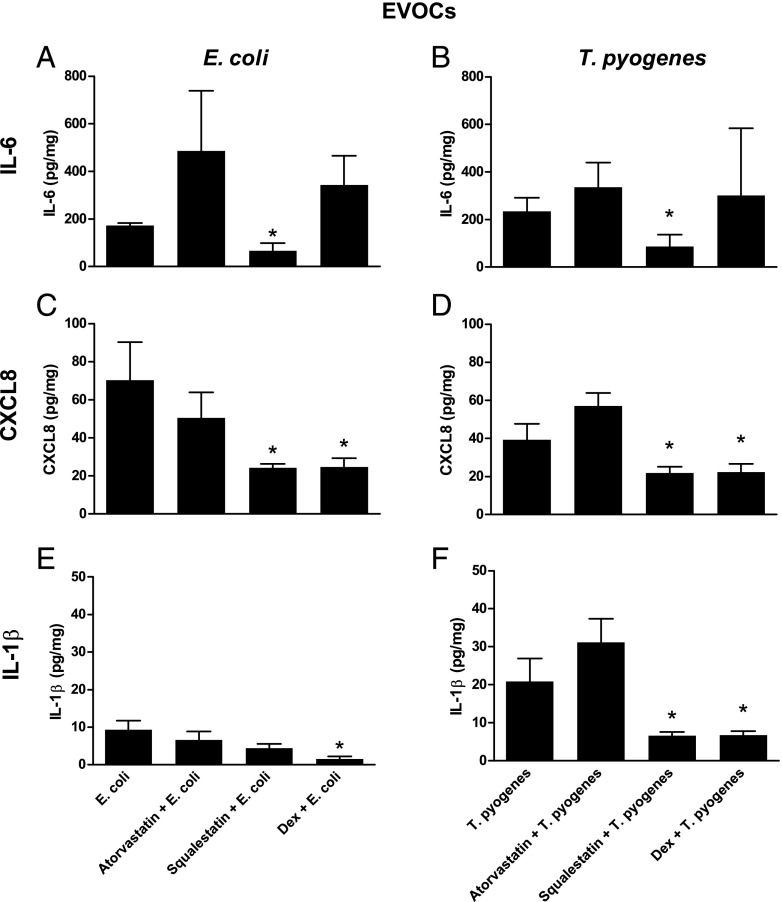
Modulating cholesterol homeostasis impacts the inflammatory response of endometrium to bacteria. Endometrial EVOCs were treated with control medium or medium containing atorvastatin (10 μM), squalestatin (10 μM), or dexamethasone (25 nM, Dex) for 24 h and then challenged with control medium or 1 × 10^3^ CFU/ml live *E. coli* or 1 × 10^8^ CFU/ml live *T. pyogenes* for 24 h. Supernatants were collected for analysis of IL-6 (**A** and **B**), CXCL8 (**C** and **D**), and IL-1β (**E** and **F**) by ELISA. Data are presented as mean (SEM) from 10 independent experiments. Data were analyzed by ANOVA and Dunnett’s pairwise multiple comparison *t* test. Values differ from *E. coli* or *T. pyogenes* treatment, *p < 0.05.

To further explore the effect of differential targeting of cholesterol biosynthesis on inflammatory responses, we used EVOCs of endometrium and live pathogenic bacteria isolated from clinical cases of endometrial disease (Fig. 3) (12, 15, 25). As expected, EVOCs challenged with 1 × 10^3^ CFU/ml live *E. coli* accumulated more IL-6 (control versus *E. coli* 11.6 ± 3.9 versus 172.9 ± 10.8 pg IL-6 per mg tissue, *p* < 0.05), CXCL8 (control versus *E. coli* 0.4 ± 0.2 versus 70.3 ± 19.9 pg/mg CXCL8, *p* < 0.05), and IL-1β (control versus *E. coli* 3.3 ± 1.0 versus 9.3 ± 3.0 pg/mg IL-1β, *p* < 0.05). However, pretreatment of EVOCs for 24 h with squalestatin reduced the accumulation of IL-6 (*p* < 0.05; Fig. 3A), whereas squalestatin and dexamethasone reduced the accumulation of CXCL8 (*p* < 0.05; Fig. 3C), and pretreatment with dexamethasone also reduced the accumulation of IL-1β (*p* < 0.05; Fig. 3E). Atorvastatin had no significant effect on inflammatory responses to live *E. coli*. Challenge of EVOCs with 1 × 10^8^ CFU/ml live *T. pyogenes* stimulated the production of more IL-6 (control versus *T. pyogenes* 11.6 ± 3.9 versus 234.3 ± 57.0 pg/mg IL-6, *p* < 0.05), CXCL8 (control versus *T. pyogenes* 0.4 ± 0.2 versus 39.3 ± 8.3 pg/mg IL-6, *p* < 0.05), and IL-1β (control versus *T. pyogenes* 3.3 ± 1.0 versus 20.9 ± 6.1 pg/mg IL-1β, *p* < 0.05). Pretreating EVOCs with atorvastatin for 24 h before challenge had no significant effect on subsequent IL responses to live *T. pyogenes*. However, pretreatment with squalestatin reduced the accumulation of IL-6 (*p* < 0.05; Fig. 3B), CXCL8 (*p* < 0.05; Fig. 3D), and IL-1β (*p* < 0.05; Fig. 3F). Taken together, these data indicate that squalestatin was most effective at limiting inflammatory responses to live bacteria or LPS in the endometrium, with a similar level of effect to the positive control dexamethasone.

### Cellular cholesterol concentration and inflammatory responses to LPS

**FIGURE 4.**
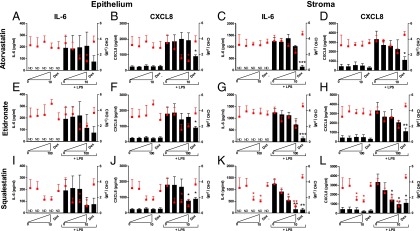
Endometrial cell inflammatory responses to LPS are attenuated by blocking FDFT1, but not FDPS or HMGCR. Endometrial epithelial (**A**, **B**, **E**, **F**, **I**, and **J**) and stromal (**C**, **D**, **G**, **H**, **K**, and **L**) cells were treated with control medium of medium containing atorvastatin (0.1–10 μM), etidronate (1–100 μM), squalestatin (0.1–10 μM), or 25 nM dexamethasone for 24 h. Cells were subsequently challenged with control medium or 100 ng/ml LPS for a further 24 h. Supernatants were collected for analysis of IL-6 (A, C, E, G, I, and K) and CXCL8 (B, D, F, H, J, and L) by ELISA (bars), and cells were lysed and stored in RIPA buffer for analysis of cholesterol (CHO) concentration by enzymatic assay (red squares). Data are presented as mean (SEM) from four independent experiments. Data were analyzed by ANOVA and Dunnett’s pairwise multiple comparison *t* test; values differ from LPS (0 + LPS), *p < 0.05, **p < 0.01, ***p < 0.001; or values differ from control (0), *p < 0.05, **p < 0.01. ND, not detected.

Based on the contrasting responses to atorvastatin and squalestatin, which act at different points in the mevalonate pathway, we reasoned that metabolites of mevalonate were likely responsible for the differing impact on the cytokine and chemokine responses. Therefore, to elucidate which molecules might alter inflammatory responses, we inhibited the mevalonate pathway in endometrial cells at three biologically relevant points: 1) HMGCR, 2) FDPS, and 3) FDFT1 (Fig. 1), using atorvastatin, etidronate, and squalestatin, respectively (Fig. 4). The effectiveness of the mevalonate pathway inhibitors used was first examined by measuring endometrial cell cholesterol content. Treatment for 24 h with the higher concentrations of atorvastatin (Fig. 4A, 4B) or squalestatin (Fig. 4I, 4J) reduced total epithelial cell cholesterol in the presence of LPS (*p* < 0.05), whereas the FDPS inhibitor, etidronate, had no significant effect on cholesterol concentrations (Fig. 4E, 4F). Stromal cell cholesterol was reduced by 100 μM etidronate in the presence of LPS (*p* < 0.05; Fig. 4G, 4H), and by 1 or 10 μM squalestatin in the presence or absence of LPS (*p* < 0.05; Fig. 4K, 4L), although not by atorvastatin (Fig. 4C, 4D). As in our previous experiments, endometrial cells secreted IL-6 and CXCL8 in response to challenge with 100 ng/ml LPS (p < 0.001; Fig. 4), and pretreatment with atorvastatin for 24 h before LPS challenge had no effect on IL-6 or CXCL8 secretion by epithelial (Fig. 4A, 4B) or stromal cells (Fig. 4C, 4D). Similarly, pretreatment for 24 h with etidronate did not impact IL-6 or CXCL8 secretion from epithelial (Fig. 4E, 4F) or stromal cells (Fig. 4G, 4H). However, pretreatment with squalestatin for 24 h reduced CXCL8 responses to LPS challenge in epithelial cells (*p* < 0.05; Fig. 4J), and IL-6 (*p* < 0.01; Fig. 4K) and CXCL8 (*p* < 0.05; Fig. 4L) responses to LPS challenge in stromal cells. These data imply that total cellular cholesterol concentrations may influence cytokine and chemokine responses to LPS.

**FIGURE 5.**
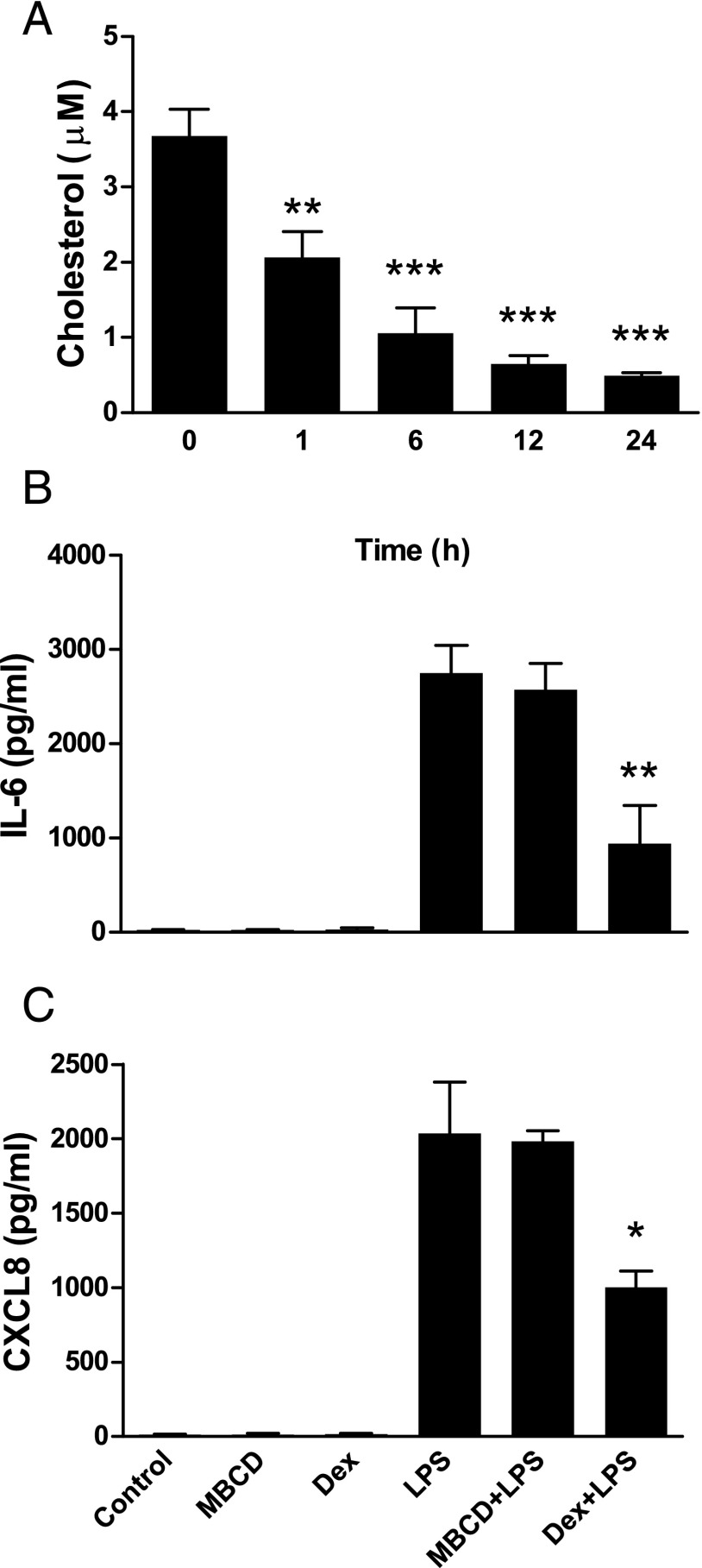
Reduced total cell cholesterol is not responsible for the modulation of LPS-mediated inflammation. Endometrial stromal cells were treated with medium containing methyl-β cyclodextrin (1 mM) for 0, 1, 6, 12, or 24 h, and cells were lysed and stored in RIPA buffer for analysis of total cell cholesterol concentration by enzymatic assay (**A**). Endometrial stromal cells were treated with control medium or medium containing methyl-β cyclodextrin (MBCD; 1 mM) or dexamethasone (Dex; 25 nM) for 24 h before challenge with control medium or 100 ng/ml LPS for a further 24 h. Supernatants were collected for analysis of IL-6 (**B**) and CXCL8 (**C**) by ELISA. Data are presented as mean (SEM) from four independent experiments. Data were analyzed by ANOVA and Dunnett’s pairwise multiple comparison *t* test; values differ from 0 h, or LPS treatment, *p < 0.05, **p < 0.01, ***p < 0.001.

To further investigate the potential role of cholesterol, we treated endometrial stromal cells with atorvastatin, etidronate, or squalestatin for 48 h and measured total cell cholesterol concentrations at several time points (Supplemental Fig. 1). Squalestatin significantly reduced total cell cholesterol by 18 h with a 62% reduction by 24 h (*p* < 0.05; Supplemental Fig. 1C). In contrast, atorvastatin only reduced cholesterol concentrations after 48 h (*p* < 0.05; Supplemental Fig. 1A), and etidronate only tended to reduce cholesterol by 48 h (*p* = 0.07; Supplemental Fig. 1B). To ascertain whether the cellular cholesterol reduction was specific for squalestatin, we treated stromal cells with an alternative FDFT1 inhibitor, CP34086894, which also significantly reduced cholesterol concentrations by 18 h, with a 58% reduction by 24 h (*p* < 0.05; Supplemental Fig. 1D). To determine whether reduced cell cholesterol may be causally associated with reduced inflammatory responses to LPS, we used methyl-β cyclodextrin, which sequesters cholesterol molecules from the cell membrane, to rapidly reduce stromal cell cholesterol concentrations by a mechanism that does not involve inhibiting the mevalonate pathway (26). Endometrial stromal cells treated with 1 mM methyl-β cyclodextrin had significantly lower cholesterol after 1-h treatment, and the concentration was 87% lower by 24 h (*p* < 0.001; Fig. 5A). However, pretreatment with methyl-β cyclodextrin for 24 h had no effect on IL-6 (Fig. 5B) or CXCL8 (Fig. 5C) secretion after challenge with LPS. To address whether cellular cholesterol reduction might affect cell health, we assessed cell viability using the MTT assay and the number of cells using the CyQUANT assay (Supplemental Fig. 2). Treatment of endometrial stromal cells with atorvastatin (0.05–10 μM; Supplemental Fig. 2A, 2B), etidronate (5–200 μM; Supplemental Fig. 2C, 2D), squalestatin (0.5–20 μM; Supplemental Fig. 2E, 2F), or methyl-β cyclodextrin (50–2000 μM; Supplemental Fig. 2G, 2H) for 24 h had no significant detrimental effect on cell viability or cell survival. Taken together these data indicate that changes in cellular cholesterol concentrations alone did not account for how manipulation of the mevalonate pathway modulated inflammatory responses to LPS.

### Isoprenoids and inflammation in the bovine endometrium

**FIGURE 6.**
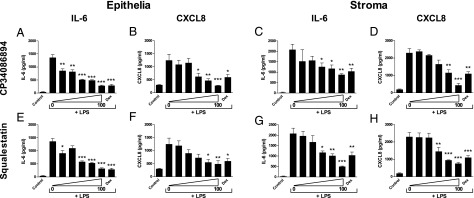
Endometrial cell inflammatory responses to LPS are attenuated in a dose-dependent manner by inhibition of FDFT1. Endometrial epithelial (**A**, **B**, **E**, and **F**) and stromal (**C**, **D**, **G**, and **H**) cells were treated with control medium or medium containing CP-34086894 [(A–D) 0.01–100 μM], squalestatin [(E–H) 0.01–100 μM], or dexamethasone (Dex; 25 nM) for 24 h. After treatment, cells were challenged with control medium or 100 ng/ml LPS for 24 h. Supernatants were collected for analysis of IL-6 (A, E, C, and G) and CXCL8 (B, F, D, and H) by ELISA. Data are presented as mean (SEM) from four independent experiments. Data were analyzed by ANOVA and Dunnett’s pairwise multiple comparison *t* test; values differ from LPS treatment, *p < 0.05, **p < 0.01, ***p < 0.001.

**FIGURE 7.**
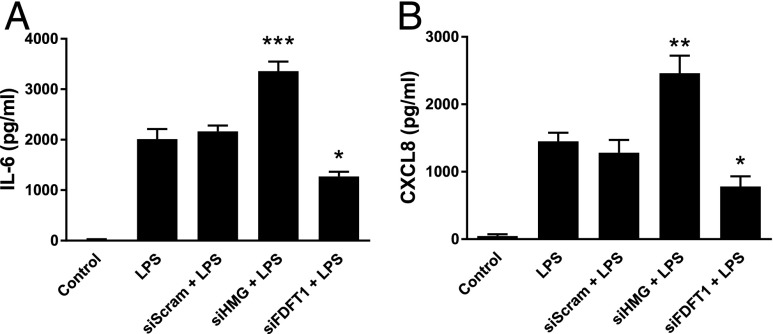
RNA interference of *HMGCR* or *FDFT1* modulates endometrial stromal cell innate immune responses to LPS. Endometrial stromal cells were transfected with scrambled siRNA (siScram) or siRNA targeting *HMGCR* (siHMG) or *FDFT1* (siFDFT1) for 48 h. After transfection, cells were challenged with control medium or 100 ng/ml LPS for 24 h. Supernatants were collected for analysis of IL-6 (**A**) and CXCL8 (**B**) by ELISA. Data are presented as mean (SEM) from four independent experiments. Data were analyzed by ANOVA and Dunnett’s pairwise multiple comparison *t* test; values differ from LPS treatment, *p < 0.05, **p < 0.01, ***p < 0.001.

In the absence of a direct effect of reduced cellular cholesterol concentrations on inflammation, the role of mevalonate metabolites was investigated. Extended dose range experiments determined that the inhibition of FDFT1 by either CP34086894 (Fig. 6A–D) or squalestatin (Fig. 6E–H) potently reduced endometrial epithelial (*p* < 0.05; Fig. 6A, 6B, 6E, 6F) and stromal (*p* < 0.05; Fig. 6C, 6D, 6G, 6H) cell IL-6 and CXCL8 responses to LPS. The data presented in Figs. 4 and 6 indicate that reduced cytokine and chemokine secretion is particularly associated with inhibition of the mevalonate pathway at the level of FDFT1, rather than HMGCR or FDPS. To provide evidence that the effects of inhibition of the mevalonate pathway were not due to off-target effects of the inhibitors used earlier, we treated endometrial stromal cells with siRNA targeting *HMGCR* (siHMG) and *FDFT1* (siFDFT1). The effectiveness of siRNA treatment was confirmed using qPCR and Western blot (Supplemental Fig. 3). Treatment of endometrial stromal cells with siHMG and siFDFT1 reduced *HMGCR* and *FDFT1* mRNA by 52 and 42%, respectively (Supplemental Fig. 3A, 3B), and protein abundance was also reduced (Supplemental Fig. 3C, 3D). Pretreatment of endometrial stromal cells with siFDFT1 reduced IL-6 (*p* < 0.05; Fig. 7A) and CXCL8 (*p* < 0.05; Fig. 7B) secretion after challenge with LPS. Conversely, pretreatment with siHMG increased IL-6 (*p* < 0.001; Fig. 7A) and CXCL8 (*p* < 0.01; Fig. 7B) secretion after challenge with LPS.

**FIGURE 8.**
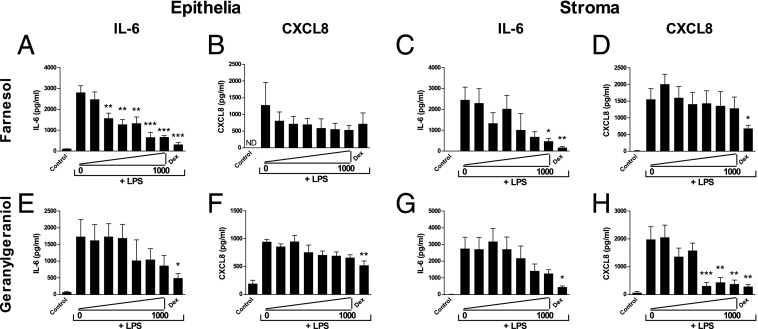
Endometrial cell inflammatory responses to LPS are attenuated in a dose-dependent manner by the addition of isoprenoids. Endometrial epithelial (**A**, **B**, **E**, and **F**) and stromal (**C**, **D**, **G**, and **H**) cells were treated with control medium or medium containing farnesol [(A–D) 0.01–1000 μM], geranylgeraniol [(E–H) 0.01–1000 μM], or dexamethasone (Dex; 25 nM) for 24 h. After treatment, cells were challenged with control medium or 100 ng/ml LPS for 24 h. Supernatants were collected for analysis of IL-6 (A, E, C, and G) and CXCL8 (B, F, D, and H) by ELISA. Data are presented as mean (SEM) from four independent experiments. Data were analyzed by ANOVA and Dunnett’s pairwise multiple comparison *t* test; values differ from LPS treatment, *p < 0.05, **p < 0.01, ***p < 0.001. ND, not detected.

Because inhibition of FDFT1 will increase the concentration of the isoprenoids farnesyl diphosphate (FPP) and geranylgeranyl diphosphate (GGPP), they might be important regulators of innate immunity. To examine this concept, we treated endometrial cells with farnesol and geranylgeraniol isoprenoid alcohols, which are converted intracellularly to their respective pathway intermediate diphosphates, FPP and GGPP (27, 28). Pretreatment of endometrial cells for 24 h with farnesol (Fig. 8A–D) or geranylgeraniol (Fig. 8E–H) reduced IL-6 and CXCL8 responses to subsequent challenge with LPS. In particular, the pretreatment of endometrial epithelial (*p* < 0.1; Fig. 8A) or stromal (*p* < 0.05; Fig. 8C) cells with farnesol for 24 h significantly reduced the accumulation of IL-6 in response to challenge with LPS (*p* < 0.05), and pretreatment of stromal cells with geranylgeraniol for 24 h significantly reduced the accumulation of CXCL8 (*p* < 0.01; Fig. 8H). Together, the use of inhibitors, siRNA, and agonists supports the concept that innate immunity is modulated by isoprenoids.

## Discussion

Evidence is emerging that integration of innate immunity, lipid metabolism, and cholesterol biosynthesis impact the development of uterine disease (8, 9). This study used primary isolated bovine endometrial cells and EVOCs as well-characterized models of endometritis (7, 15), to explore cellular pathways that modulate inflammatory responses within the endometrial tissue. Of particular interest were differential inflammatory responses after targeting of enzymes within the mevalonate pathway. Inhibition of the rate-limiting enzyme HMGCR by statins had little effect on cellular inflammatory responses, but inhibition of the enzyme FDFT1 was as effective as using the standard anti-inflammatory, dexamethasone. There was evidence that the reduced inflammation was not a consequence of lower cellular cholesterol, but rather mediated by isoprenoids.

Inhibition of FDFT1 by squalestatin or CP34086894 was equally effective at reducing the inflammatory response to bacteria in organ cultures, or to LPS in epithelial or stromal cells. In addition, siRNA targeting FDFT1 reduced LPS-mediated IL-6 and CXCL8 secretion by endometrial cells. The more rapid and efficient reduction in cholesterol within cells treated with FDFT1 inhibitors, compared with atorvastatin or etidronate, implied that cellular cholesterol may impact innate immune responsiveness to LPS. However, a similar reduction of cholesterol using methyl-β cyclodextrin had no effect on LPS-mediated IL-6 secretion, suggesting that cellular cholesterol was not the determining factor for modulating innate immunity. Indeed, cholesterol reduction per se is also not the main mechanism of statin-driven immune modulation, which occurs primarily via disrupted intracellular signaling and trafficking brought about by a reduction of intracellular cholesterol intermediates (29).

In this study, inhibition of HMGCR either had no effect or increased LPS-mediated endometrial innate immune responses. The implication in this study is that molecules downstream from mevalonate are capable of regulating endometrial innate immune responses to LPS. At high concentrations, statins reduce the production of isoprenoids, including FPP and GGPP, in various cell types through depletion of isoprenoid precursors within the mevalonate pathway (29–33). The isoprenoids FPP and GGPP are essential for the posttranslational modification, membrane attachment, and biological activity of Ras-family G proteins and Rho GTPases, respectively (34, 35). Therefore, their depletion within the cell affects a wide range of cellular pathways, as indeed does their excessive accumulation (36, 37). In humans, the effect of manipulating isoprenoid availability on LPS responsiveness is exemplified by reduced TLR4 expression on monocytes isolated from patients treated with statins for 4 wk. Reduced TLR4 expression and LPS-mediated inflammatory responses are mediated via inhibition of protein geranylgeranylation and farnesylation, further demonstrating the importance of the interaction between cholesterol biosynthesis and innate immunity (33).

Inhibition of FDFT1, by inhibitors or siRNA, increases intracellular concentrations of FPP and GGPP (36, 37). Furthermore, the isoprenoid alcohols, farnesol and geranylgeraniol, both of which are converted to their respective pathway intermediate diphosphates, FPP and GGPP (27, 28), attenuated endometrial cell IL-6 and CXCL8 responses to LPS in this study. The anti-inflammatory effect of isoprenoids has previously been demonstrated in a mouse model of mevalonate kinase deficiency, a rare disorder characterized by recurrent inflammatory episodes. Systemic inflammatory responses, induced by the administration of muramyldipeptide and the aminobisphosphonate alendronate, were inhibited by the addition of exogenous isoprenoids demonstrating an immune-regulatory function (38). Furthermore, the inflammatory phenotype of mevalonate kinase deficiency in humans is specifically driven by a lack of mevalonate-derived isoprenoids (39). The implication is that isoprenoids are involved in the regulation of inflammation, and our data demonstrate that FPP and GGPP regulate inflammation within the endometrium. The basis for this regulation remains unclear, and elucidation of the molecular mechanisms involved requires further work, which will form the foundation of future studies.

In summary, we show that manipulating the mevalonate pathway modulates innate immunity in endometrial cells and tissue. Surprisingly, inhibition of FDFT1 (squalene synthase) reduced inflammatory responses to bacteria or LPS in contrast with modulating the rate-limiting enzyme, HMGCR, with statins, which had no beneficial effect. These findings are important because they uncover a role for FDFT1 and geranyl and farnesyl isoprenoids in mucosal immunity. Furthermore, these findings could be translated to use topical administration of FDFT1 inhibitors into the uterus to limit the severity of bacterial endometritis.

## Supplementary Material

Data Supplement
