# Supplementary material for: Mevalonate Biosynthesis Intermediates Are Key Regulators of Innate Immunity in Bovine Endometritis
Source: J Immunol. 2015 Dec 16;196(2):823–31. doi: 10.4049/jimmunol.1501080 (PMC4705593; doi:10.4049/jimmunol.1501080)
Supplement: Data Supplement [file JI_1501080.zip › JI_1501080_Supplemental_Material_1.pdf]

Supplemental Table I

Small molecules screened

Name	Formula	MW	Mode of action	Supplier	IL-6 ± SEM (pg/ml)	Cell Viability ± SEM (% control)
PF-03052334-02	Not available	224.9	HMG CoA reductase (antagonist)	Pfizer	1801.2 ± 599.1	148.3 ± 15.5
PF-00261809-02	Not available	212.1	HMG CoA reductase (antagonist)	Pfizer	732.2 ± 190.8	71.9 ± 31.5
PF-02887172-02	Not available	220.4	HMG CoA reductase (antagonist)	Pfizer	691.2 ± 209.2	88.8 ± 29.5
PF-03591518-02	Not available	188.13	HMG CoA reductase (antagonist)	Pfizer	675.0 ± 186.1	83.5 ± 27.6
PF-03491165-00	Not available	214.2	HMG CoA reductase (antagonist)	Pfizer	827.1 ± 239.4	83.9 ± 29.0
PF-03201237-02	Not available	177.8	HMG CoA reductase (antagonist)	Pfizer	791.1 ± 224.7	85.3 ± 31.0
Atorvastatin	$C_{33}H_{35}FN_2O_5$	558.64	HMG CoA reductase (antagonist)	Pfizer	3112.9 ± 875.1	101.1 ± 9.4
Squalestatin	$C_{35}H_{46}O_{14}$	690.73	FDFT1 (antagonist)	Glaxo SmithKline	408.7 ± 86.4	88.0 ± 31.2
CP-340868-94	$C_{32}H_{35}ClN_2O_4S$	579.14	FDFT1 (antagonist)	Pfizer	287.9 ± 43.0	222.0 ± 7.1
TO901317	$C_{17}H_{12}NSO_3F_9$	481.3	LXR (oxysterol receptor) (agonist)	Sigma-Aldrich	828.4 ±89.9	225.9 ± 16.4
Alpha cyclodextrin	$C_{36}H_{60}O_{30}$	972.84	Cholesterol sequester	Sigma-Aldrich	930.6 ± 256.5	100.8 ± 25.7
Beta cyclodextrin	$C_{42}H_{70}O_{35}$	1134.98	Cholesterol sequester	Sigma-Aldrich	952.9 ± 288.4	105.8 ± 5.7
Methyl-beta cyclodextrin	$C_{56}H_{98}O_{35}$	1310	Cholesterol sequester	Sigma-Aldrich	1009.4 ± 263.0	110.9 ± 12.0
Gamma cyclodextrin	$C_{48}H_{80}O_{40}$	1297.12	Cholesterol sequester	Sigma-Aldrich	1140.3 ± 330.0	101.4 ± 7.4
Resolvin D1	$C_{22}H_{32}O_5$	376.5	Resolvin D1 receptor (antagonist)	Cayman Chemical	784.0 ± 44.1	81.9 ± 3.4
Lipoxin A4	$C_{20}H_{32}O_5$	352.5	Lipoxin A4 receptor (antagonist)	Cayman Chemical	804.2 ± 44.7	90.6 ± 22.2
CP-481715	$C_{26}H_{31}FN_4O_4$	482.54	Chemokine receptor 1 (antagonist)	Pfizer	437.5824 ± 185.2	162.6 ± 42.4
865569	Not available	-	Chemokine receptor 1 (antagonist)	Pfizer	666.9 ± 98.3	177.9 ± 39.3
04136309-00	Not available	-	Chemokine receptor 2 (antagonist)	Pfizer	507.0 ± 197.4	188.1 ± 34.6
04973563-00	Not available	-	Chemokine receptor 5 (antagonist)	Pfizer	607.5 ± 94.2	186.9 ± 36.1
00227153-00	Not available	-	Chemokine receptor 5 (antagonist)	Pfizer	732.6 ± 105.7	178.8 ± 29.2
230988-A	Not available	-	Chemokine receptor 2/5 (antagonist)	Pfizer	786.0 ± 87.6	135.9 ± 22.7
04178903-00	Not available	-	Chemokine receptor 2/5 (antagonist)	Pfizer	604.2 ± 154.2	180.7 ± 31.1
04254196-18	Not available	-	Chemokine receptor 2/5 (antagonist)	Pfizer	875.0 ± 117.8	158.3 ± 35.0
Dexamethasone	$C_{22}H_{29}FO_5$	392.46	Glucocorticoid receptor (agonist)	Sigma-Aldrich	304.9 ± 49.2	141.9 ± 12.6
Oestradiol	$C_{18}D_3H_{21}O_2$	272.38	Oestrogen receptor (agonist)	Sigma-Aldrich	901.547 ± 107.4	107.1 ± 8.7
Progesterone	$C_{21}H_{30}O_2$	314.46	Progesterone receptor (agonist)	Sigma-Aldrich	868.4 ± 201.5	92.0 ± 4.9

ERB-041	$C_{15}H_{10}FNO_3$	271.24	Oestrogen receptor beta (agonist)	Pfizer	930.2 ± 134.2	190.1 ± 9.5
2,3-Bis(4-hydroxyphenyl)propionitrile	$C_{15}H_{13}NO_2$	239.27	Oestrogen receptor beta (agonist)	Sigma-Aldrich	899.3 ± 149.6	231.4 ± 20.1
1,3,5-Tris(4-hydroxypheyl)-4-propyl-1H-pyrazole	$C_{24}H_{22}N_2O_3$	386.44	Oestrogen receptor alpha (agonist)	Sigma-Aldrich	1054.2 ± 87.8	178.7 ± 19.1
1,3-Bis(4-hydroxyphenyl)-4-methyl-5-[4-(2-piperidinylethoxy)phenol]-1H-pyrazole dihydrochloride hydrate	$C_{29}H_{31}N_3O_3$	542.50	Oestrogen receptor alpha (antagonist)	Sigma-Aldrich	995.1 ± 142.9	204.9 ± 17.1
Mifepristone (RU-486)	$C_{29}H_{35}NO_2$	429.59	Progesterone and glucocorticoid receptor (antagonist)	Sigma-Aldrich	944.0 ± 174.6	296.1 ± 38.1
PF-02413873-00	$C_{18}H_{21}N_3O_3S$	359.44	Progesterone receptor (antagonist)	Pfizer	821.1 ± 135.7	296.7 ± 54.3
PF-03491390	$C_{26}H_{27}F_4N_3O_7$	569.50	Pan-caspase (antagonist)	Pfizer	856.2 ± 97.3	188.9 ± 34.3
CE-224535	$C_{22}H_{29}ClN_4O_6$	480.94	P2X7 receptor (antagonist)	Pfizer	726.5482 ± 157.3	179.9 ± 38.6
PF184	$C_{32}H_{32}ClFN_6O_4$	619.09	IkappaB kinase complex (antagonist)	Tocris Bioscience	769.0 ± 195.4	220.4 ± 11.0
IRAK4	Not available	-	IRAK4 inhibitor	Pfizer	108.1 ± 17.3	152.1 ± 32.4
IKK	Not available	-	IKK inhibitor	Pfizer	163.6 ± 10.8	96.9 ± 24.3
MEK	Not available	-	MEK inhibitor	Pfizer	155.4 ± 36.2	123.0 ± 18.1
P38 733836	Not available	-	p38 inhibitor	Pfizer	215.9 ± 43.5	97.9 ± 8.7
P38 709733	Not available	-	p38 inhibitor	Pfizer	301.8 ± 80.1	132.2 ± 3.0
Tpl2 199727	Not available	-	Tpl2 kinase inhibitor	Pfizer	245.7 ± 57.2	115.9 ± 7.9
Tpl2 397103	Not available	-	Tpl2 kinase inhibitor	Pfizer	206.8 ± 79.4	149.3 ± 21.1
JAK	Not available	-	JAK inhibitor	Pfizer	222.8 ± 69.0	115.8 ± 19.8
STATVI	$C_{16}H_{15}NO_7S$	365.36	STAT3 inhibitor	-	242.6 ± 178.7	73.7 ± 12.4
STATTIC	$C_8H_5NO_4S$	211.2	STAT3 inhibitor	Merck Millipore	353.4 ± 184.3	66.9 ± 6.2
ULHA	Not available	-	-	-	145.7 ± 32.3	102.8 ± 1.1
HHA	Not available	-	-	-	256.3 ± 38.9	111.9 ± 4.6

Supplemental figure 1

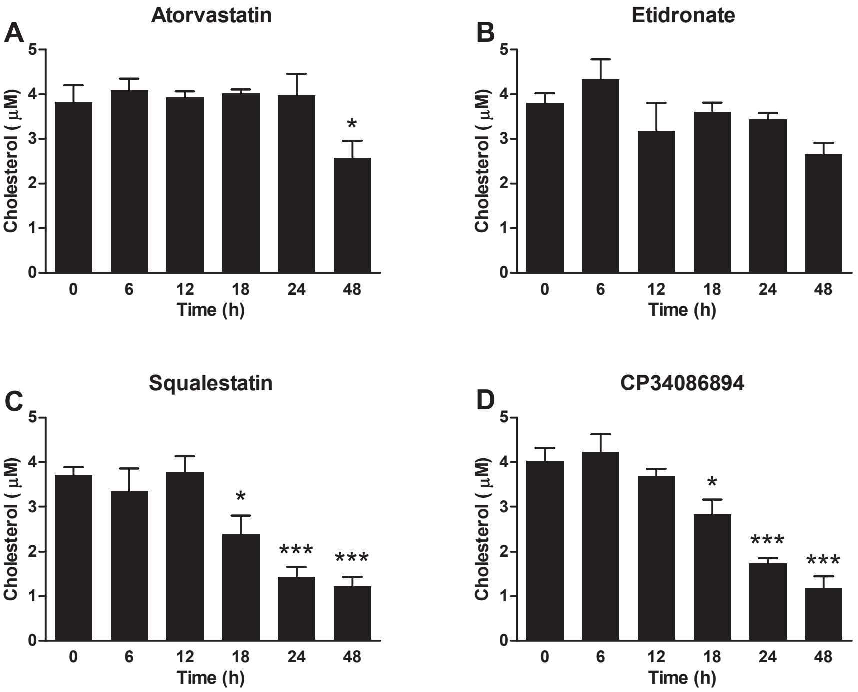

Inhibition of FDFT1 reduces total cell cholesterol more efficiently than inhibition of HMGCR or FDPS. Endometrial stromal cells were treated with medium containing 10 µM Atorvastatin, 100 µM Etidronate, 10 µM Squalestatin or 10 µM CP34086894 for 0, 6, 12, 18, 24 or 48 h. After treatment, supernatants were discarded and cells lysed and stored in RIPA buffer for analysis of total cell cholesterol by enzymatic assay. Data are presented as mean (SEM) from four independent experiments. Data were analysed by ANOVA and Dunnett's pairwise multiple comparison t-test; values differ from 0 h, * $P < 0.05$, *** $P < 0.001$.

Supplemental figure 2

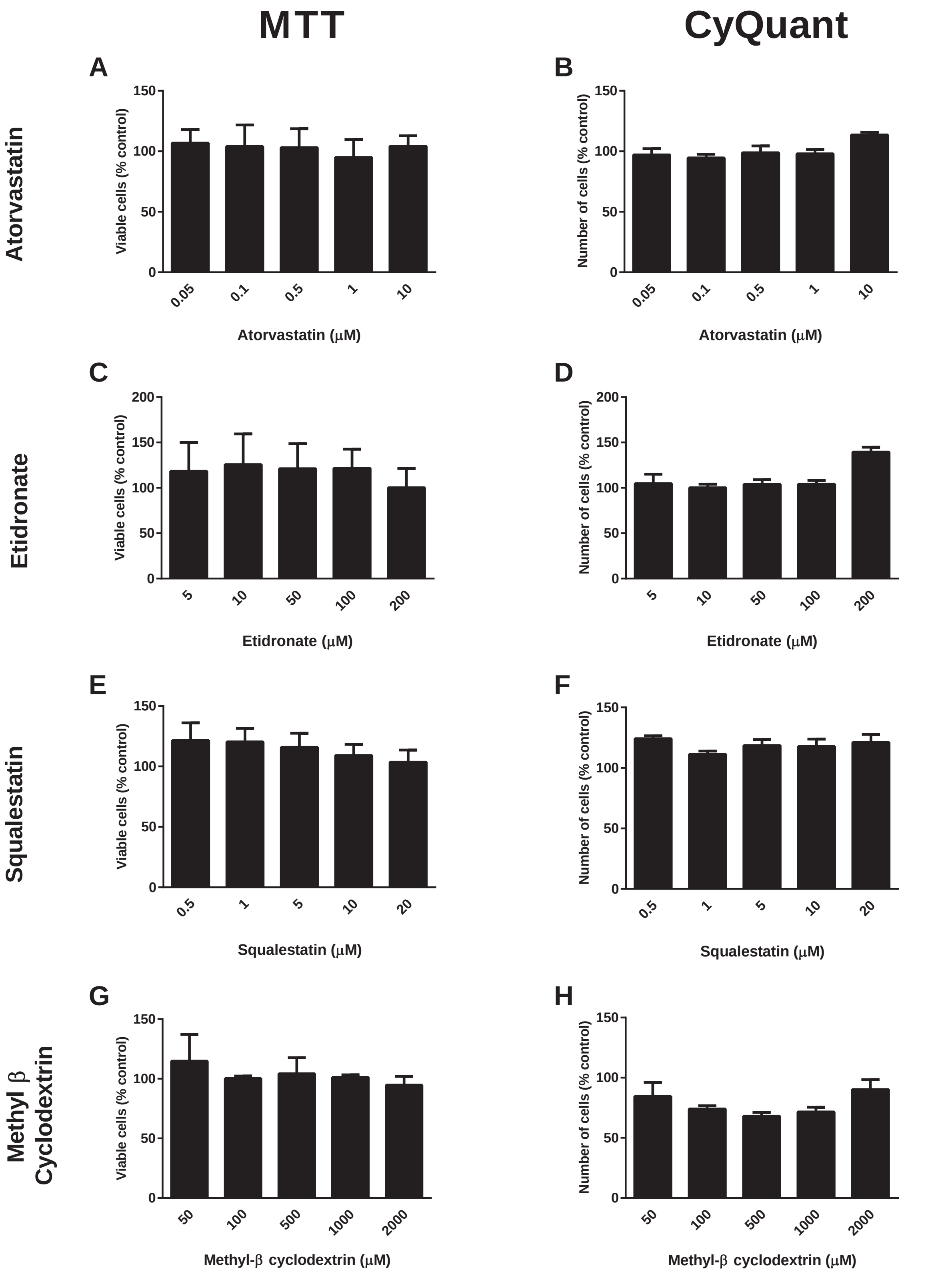

Cellular cholestreol reduction did not affect cell health. Endometrial stromal cells were treated with Atorvastatin (0.05 - 10 μM), Etidronate (5 - 200 μM), Squalestatin 0.5 - 20 μM) or Methyl-β Cyclodextrin (50 - 2000 μM) for 24 h. Supernatants were removed and cell viability assessed by MTT assay as a percent of control (A, C, E, G). At the end of the assay the number of cells were measured as a percent of control using the CyQUANT assay (B, D, F, H). Data are presented as mean (SEM) from four indepenednt experiments. Data were analysed by ANOVA and Dunnett's pairwise multiple comparison t-test; values do not differ from control, P > 0.05.

Supplemental figure 3

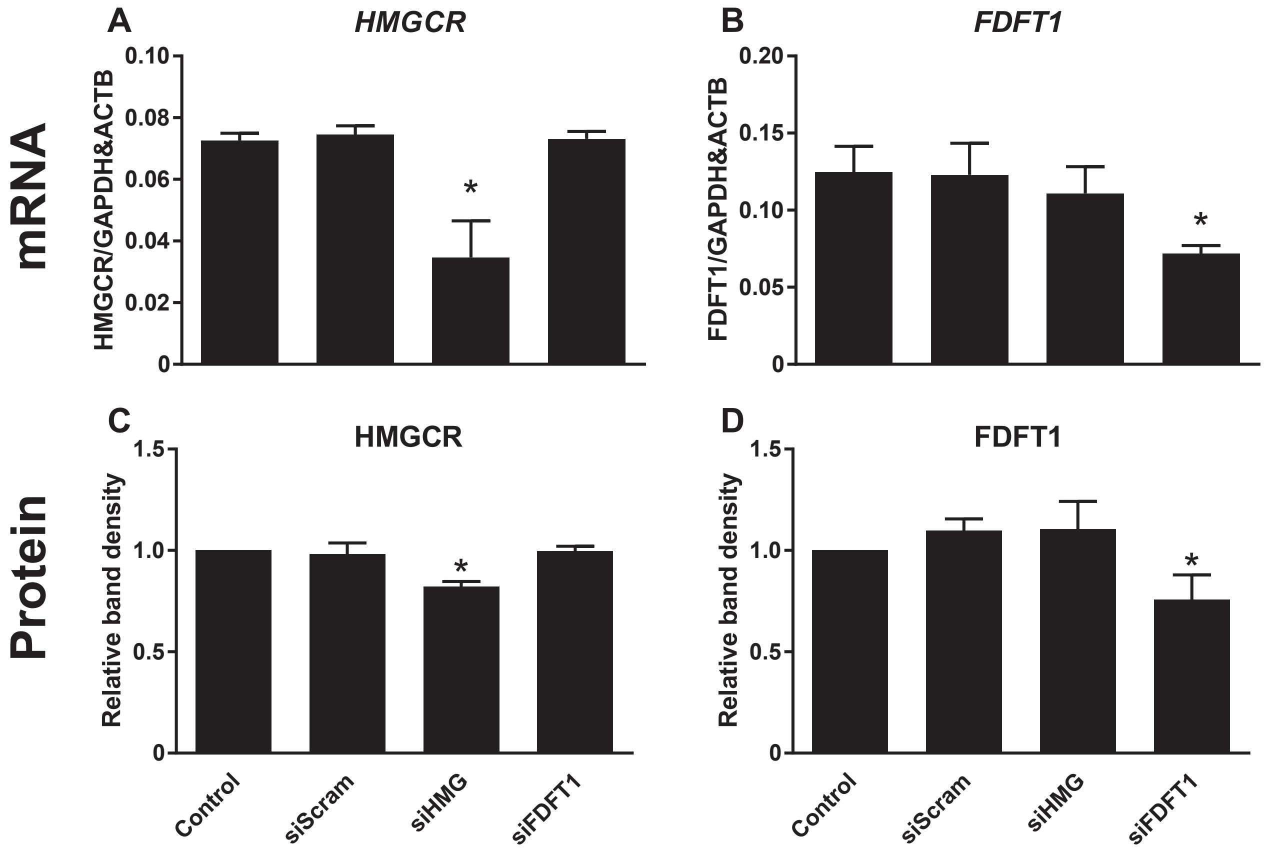

Treatment of endometrial stromal cells with siRNA attenuated the expression of target RNA and protein. Endometrial stromal cells were transfected with control scrambled siRNA (siScram) or siRNA targeting HMGCR (siHMG) or FDFT1 (siFDFT1). Supernatants were removed and cells lysed and stored in RLT buffer at -80°C for analysis of *HMGCR* and *FDFT1* by quantitative PCR (A, B), or RIPA buffer at -80°C for analysis of HMGCR and FDFT1 by western blot (C, D). Data presented as mean (SEM) from three independent experiments. Data were analysed by ANOVA and Dunnett's pairwise multiple comparison t-test; values differ from control, * $P < 0.05$, ** $P < 0.01$.
